# After the fire: A qualitative study of the role of long-term recovery organizations in addressing rural communities’ post-wildfire needs

**DOI:** 10.1088/2752-5309/acd2f7

**Published:** 2023-05-30

**Authors:** Kathleen Moloney, Jamie Vickery, Jeremy Hess, Nicole Errett

**Affiliations:** 1 Department of Environmental and Occupational Health Sciences, School of Public Health, University of Washington, Seattle, WA, United States of America; 2 Department of Global Health, School of Public Health, University of Washington, Seattle, WA, United States of America; 3 Department of Emergency Medicine, School of Medicine, University of Washington, Seattle, WA, United States of America; 4 Center for Health and the Global Environment (CHanGE), School of Public Health, University of Washington, Seattle, WA, United States of America; 5 Department of Health Systems and Population Health, University of Washington, Seattle, WA, United States of America

**Keywords:** wildfire, long-term recovery, disaster, health equity

## Abstract

U.S. wildfire activity has increased over the past several decades, disrupting the systems and infrastructure that support community health and resilience. As the cumulative burden of wildfire damage is projected to increase, understanding an effective community recovery process is critically important. Through qualitative interviews with leaders of long-term recovery organizations (LTROs), a key component of wildfire recovery, we explored barriers and facilitators to LTROs’ ability to support post-wildfire needs among rural communities. Between February-May 2022, we conducted surveys and semi-structured interviews with 18 leaders from six LTROs serving rural communities in Washington, Oregon, and California impacted by wildfires between 2015–2020. The Robert Wood Johnson Foundation’s Culture of Health Framework informed the semi-structured interview guide and *a priori* codebook, to examine LTROs’ ability to address post-wildfire community needs from a health equity perspective. Additional codes were added through an inductive approach, and emerging themes were identified. Our findings indicate that LTROs face many barriers in addressing community needs post-wildfire, including the policies governing access to and the slow arrival of recovery resources, the intertwined nature of community economic health and built environment restoration, and the challenge of forming a functional LTRO structure. However, participants also identified facilitators of LTROs’ work, including the ability of LTROs and their government partners to adapt policies and procedures, and close collaboration with other community organizations. Factors both internal and external to the community and LTROs’ organizational characteristics influence their ability to address community needs, essential to health, post-wildfire. This study’s findings suggest the need for policy improvements to promote more equitable recovery resource access, that economic recovery should be a core LTRO function, and that recovery planning should be incorporated into community disaster preparedness activities. Future research should focus on LTROs’ role in other contexts and in response to other disasters.

## 1. Background

U.S. wildfire activity, particularly in western states, has increased in intensity and duration over the past several decades [1–3]. In 2020 alone, nearly 59 000 U.S. wildfires occurred, cumulatively burning over 10.1 million acres [4]. Beyond the direct impacts to human health such as toxicant exposure and smoke inhalation, wildfires have massive economic and social consequences that can reverberate throughout impacted communities for years, disrupting housing, employment, and other aspects of social and economic infrastructure that support community health and wellbeing [3]. If the trend towards hotter, drier, and longer summers continues, as current and projected data suggest it will, the cumulative burden of wildfire damage is expected to increase over the coming decades [1]. Additionally, a growing number of residences are being built in areas vulnerable to wildfire damage, particularly within the wildland urban interface [5]. These combined factors indicate that understanding what constitutes an effective community recovery process after a wildfire, as well as barriers and facilitators to implementing that process, is critically important.

Wildfires often result in widespread destruction, including to the systems and infrastructure that support people’s health and wellbeing. Previous research has documented the severe, lasting impacts of wildfires, and of disasters more broadly, affecting the social and economic infrastructure that support community health and wellbeing, including economic stability, education access and quality, healthcare access and quality, neighborhood and built environment, and social and community context [3, 6, 7]. Post-disaster impacts on one domain can compound negative impacts on others. For instance, many survivors face extended difficulty securing new permanent housing post-wildfire. This housing instability in turn is associated with increased mortality [3], and families who struggle to find permanent housing post-disaster may relocate multiple times. Combined with the destruction of local schools, this housing instability disrupts children’s school attendance and eventual educational attainment [8]. The displacement of the limited local medical providers that a rural community had available pre-wildfire, as well as the destruction of local medical facilities, further curtail healthcare access [3, 6]. Those with the least resources pre-disaster are the most likely to experience lasting disruptions to their economic stability, housing, education access, and other key social and economic resources that support wellbeing, exacerbating pre-disaster inequities [9].

Beyond pre-existing community capacity, community recovery is influenced by the amount of external resources made available to a community following a disaster. Federal resources, made possible through a Major Disaster Declaration, can kickstart the disaster recovery process and address post-disaster community needs [10]. When a Major Disaster Declaration occurs, impacted communities are potentially eligible for two categories of Federal Emergency Management Agency (FEMA) assistance, depending on the capacity and financial resources of state and local government and the scale of the disaster’s impacts to community infrastructure, housing, economic resources, and human life [11]: (1) public assistance, which is financial assistance for state, tribal, and local governments and some nonprofit organizations to support emergency work and the repair or replacement of disaster-damaged facilities and/or infrastructure [12], and (2) individual assistance, which can include financial assistance and other services for individuals and households such as housing assistance, disaster case management, and mental health counseling [13]. Beyond the resources made directly available via FEMA individual and public assistance, Major Disaster Declarations typically trigger an influx of additional resources into disaster-impacted communities, such as the activation of small business administration (SBA) assistance [14] or the arrival of technical support that can facilitate the coordination of resources and transition from disaster response to long term recovery [15, 16].

While a growing body of research documents associations between the timing, amount, and accessibility of FEMA assistance and community recovery outcomes [10, 17, 18], not all communities receive Major Disaster Declarations or the full amount of support that they potentially make available. These communities rely heavily on local resources and expertise to support their community, organized through long-term recovery organizations (LTROs). In rural communities in particular, where social service delivery infrastructure may be limited pre-disaster, LTROs serve a community’s recovery needs post-disaster [19], ideally providing a coordinated entry point where impacted community members may seek support and access to resources. In contrast to other organizations supporting disaster recovery, whose presence in a community may wane within months post-disaster, LTROs remain dedicated to the communities they originally formed to support, operating for years after the disaster. LTRO leadership may consist of community members, representatives from organizations involved in the recovery process, or a mix of both. LTROs can be structured in numerous ways, depending on the resources and needs of the communities they serve, but the typical LTRO model includes a board and/or executive director overseeing various committees, each tasked with a particular aspect of long-term recovery [20].

However, despite LTROs’ unique perspective on and centrality to long-term community recovery, little is understood about their role in meeting community recovery needs or what contextual determinants impact their ability to meet those needs. Few studies on community recovery have investigated LTROs’ role in this process, and those that have did so by primarily focusing on recovery processes in urban areas impacted by hurricanes [19, 21, 22]. Further research on LTROs is clearly needed, with a particular focus on the unique challenges faced by LTROs striving to address needs related to the social and economic fabric of rural communities that support health and wellbeing following wildfires. In response, this study explored barriers and facilitators to LTROs addressing such community needs after a wildfire disaster through qualitative interviews with LTRO leaders in wildfire-affected communities. Moreover, the study sought to identify policy solutions that can be leveraged or facilitated by LTROs to support health equity in a post-disaster context.

## 2. Method

### 2.1. Study design

We conducted a brief pre-interview survey and semi-structured interviews with 18 current or former LTRO leaders. We then coded and thematically analyzed transcripts to identify emerging themes in response to the study’s aims.

### 2.2. Sampling

We used purposive and snowball sampling to identify current and former LTRO leaders for study participation. Inclusion criteria were having held or currently holding a leadership position within an LTRO for at least six months, either as an employee or board member, and serving a rural (i.e. a population of ⩽50 000) [23] community or communities in Washington, Oregon, or California impacted by a wildfire disaster between 2015 and 2020. For the purpose of this study, a wildfire disaster is defined as an unplanned fire in a natural area [24] that destroyed ⩾200 residences and/or important community structures (e.g. schools, government buildings, and/or businesses). All study participants were over 18 years of age.

We identified LTROs meeting the inclusion criteria via a web search and existing professional networks. If direct contact information (i.e. an email address or phone number) for a current or former member of an eligible LTRO could be identified, the study team reached out directly to that LTRO member to confirm study eligibility and invite them to participate, as well as to solicit recommendations for other potential study participants from their LTRO. In the case that no direct contact information was available for an LTRO member, we sent an email and/or phone message to the general LTRO contact information provided on the LTRO’s website.

### 2.3. Data collection

The semi-structured interview guide (appendix A) included questions and prompts informed by the Robert Wood Johnson Foundation’s (RWJF) Culture of Health (COH) Framework [25], which identifies four action domains of work towards health equity. These domains incorporate aspects of social and economic infrastructure that support community health and wellbeing, as well as community social capital, cohesion, and collaboration, which have been previously shown to impact community disaster recovery trajectories [25–27]. We conducted semi-structured interviews lasting 30–90 min with study participants individually in English via video conference or telephone from 22 February to 10 May 2022. We obtained verbal consent from each participant prior to commencing the interview. All interviews were audio recorded, professionally transcribed, and reviewed to ensure transcription accuracy.

### 2.4. Data analysis

We used thematic coding to analyze interview transcripts. The health equity action areas outlined by the RWJF COH Framework [25] guided the development of *a priori* themes and codes; we used an inductive approach to build upon the initial codebook to capture additional concepts. Before finalizing the coding scheme, two team members co-coded approximately 10% of the interview transcripts using NVivo 12 qualitative analysis software to ensure reliability and validity of the qualitative codebook and its application. After merging the coding files from each coder and conducting a coding comparison query, we discussed codes below 90% agreement and revised the codebook accordingly. After the codebook was finalized (appendix B), one study team member re-coded the first two transcripts and coded the remaining 16 transcripts. After the coding process was complete, we created analysis memos for each RWJF health equity domain code, summarizing the key barriers and facilitators pertaining to each domain.

## 3. Results

### 3.1. Participant characteristics

Of the 22 current or former LTRO leaders from eight LTROs contacted, 18 participants from six LTROs agreed to participate, including at least two representatives from each LTRO. The LTROs served communities in Washington state, Oregon, and California (table 1). While all the wildfire disasters these LTROs were created in response to eventually received a Major Disaster Declaration, only four were granted both individual and public assistance from the Federal Emergency Management Agency (FEMA) [12, 13].

**Table 1.** Characteristics of participating long-term recovery organizations (LTROs) and the communities they served (*N* = 6).

**Community characteristics**	***N* (%)**
**State**	
WA	2 (33.3%)
OR	3 (50.0%)
CA	1 (16.7%)
**Year of wildfire**	
2015	1 (16.7%)
2018	1 (16.7%)
2020	4 (66.7%)
**FEMA assistance provided**	
Public Assistance Only	2 (33.3%)
Individual & Public Assistance	4 (66.7%)
**LTRO characteristics**	***N* (%)**
**Number of board members**	
Less than 5	1 (16.7%)
5–10	3 (50.0%)
11–15	1 (16.7%)
More than 15	1 (16.7%)
**Highest number of employees**	
1	3 (50.0%)
2	1 (16.7%)
3 or more	2 (33.3%)
**Composition of LTRO**	
Primarily community members	5 (83.3%)
About an equal mix of community members & disaster relief/recovery professionals	1 (16.7%)
**LTRO funding sources**	
Individual donations	6 (100.0%)
Federal recovery assistance	5 (83.3%)
Other government grants	5 (83.3%)
Grants from NGOs	6 (100.0%)
Other	3 (50%)

Five of the 18 individual study participants were wildfire survivors themselves, four of whom had lost their home in the wildfires (table 2). The majority of participants had no prior disaster relief or recovery experience before joining the LTRO (table 2). Participants described numerous barriers and facilitators to LTROs’ work to meet community needs (table 3), discussed below.

**Table 2.** Individual participant characteristics (*N* = 18).

**Characteristic**	***N* (%)**
**Survivor of wildfire**	5 (27.8%)
**Position on LTRO**	
Current board member	10 (55.6%)
Current employee	7 (38.9%)
Former employee	1 (5.6%)
**Length of service with LTRO**	
6 months–less than 1 year	4 (22.2%)
1 year–less than 2 years	8 (44.4%)
2 years–less than 3 years	1 (5.6%)
3 or more years	5 (27.8%)
**Disaster relief/recovery experience prior to LTRO service**	
No prior experience	11 (61.1%)
Less than 1 year	2 (11.1%)
1 year–less than 3 years	3 (16.7%)
3 years–5 years	1 (5.6%)
Greater than 5 years	1 (5.6%)

**Table 3.** Barriers & facilitators to LTROs’ ability to meet post-wildfire community needs.

**Theme**	**Key examples**
**Barriers**	
Policies that Govern Access to Key Recovery Resources Exacerbate Inequities	Those with informal rental or housing arrangements unable to be served by FEMA or standard disaster case management models Multigenerational households receive less FEMA assistance FEMA denial & appeal process difficult to navigate
Creating a Functional LTRO Structure Matched to Community Capacity & Needs	Interpersonal conflicts among LTRO leaders during the first 1–2 years of existence Standard LTRO model does not always match community needs
Slow Delivery of Recovery Resources	FEMA trailers can take up to 9 months post-disaster to arrive Delayed Major Disaster Declaration inhibits recovery progress Delay in other resources delays distribution of Unmet Needs Roundtable funds
The Intertwined Nature of Community Economic Health & the Built Environment	Loss of affordable housing for the workforce, and businesses cannot operate without a workforce Loss of revenue source for town leaves lack of funding to rebuild infrastructure or community spaces
**Facilitators**	
Collaboration with Partners with a Local Presence	Use of pre-existing structures to delivery recovery services Maximizes recovery funding Allows vulnerable community members to access key resources
Flexibility in Policies & Procedures Created by LTROs & Government Partners	Creating a ‘dual’ case management process that allows those without access to standard disaster case managers access to recovery resources Relaxing land use & building regulations FEMA representatives dressing in plain clothes to better engage with mixed documentation status families Activating SBA assistance prior to a Major Disaster Declaration when this declaration was delayed

### 3.2. Barriers to LTROs’ ability to address community needs

#### 3.2.1. Policies that govern access to key recovery resources exacerbate inequities

Participants described numerous ways that policies and standard procedures governing access to key recovery resources left many impacted community members unable to obtain such resources. Accessing FEMA individual assistance specifically was described as a lengthy, confusing application process that required a level of technology literacy that many community members impacted by the wildfire lacked. This process was described as not ‘trauma-informed,’ failing to treat applicants with the respect and kindness that facilitate empowered choices among those who have recently experienced a traumatic event [28]. For instance, harsh language in FEMA communication such as ‘denial’ and ‘eviction’ created alarm and obscured the ability of recipients to appeal these decisions. Additionally, FEMA’s narrowly prescribed definitions of residency, which excluded undocumented immigrants and those who had informal rental agreements or otherwise lacked required documentation, left the most vulnerable community members without access to individual assistance [13]. Even among those who received individual assistance, inequities persisted. For instance, the allocation of assistance by household, without accounting for multigenerational households, meant that many low-income and Hispanic/Latinx families received proportionally less assistance than White, wealthier households.

Policies about who qualifies for a disaster case manager under standard models [29] endorsed by FEMA provided a similar barrier. These disaster case management models applied strict definitions to residency that mirrored FEMA policies, categorizing large numbers of community members who did not meet these requirements ‘pre-disaster homeless.’ The standard disaster case management goal of returning community members to their pre-disaster state was poorly matched to the needs of those who were living in substandard housing conditions pre-disaster. In the words of one participant,

*‘We’ve got […] lots of different, unique relationships between people and how they found housing and food and sustain themselves, which has been an interesting challenge with recovery [..] when you have an entire town and all of those networks and communities gone, that’s not an easy thing to just replace. We can’t put people back in the shed in exchange for caregiving services.’*

#### 3.2.2. Creating a functional LTRO structure matched to community capacity and needs

Participants highlighted the numerous challenges of trying to structure and staff a brand-new organization after a major disaster. Interpersonal conflicts among LTRO leaders or key partners, which often led LTRO leaders to step down and/or necessitated LTRO restructuring, were an early barrier to LTRO operation. These conflicts arose from pre-existing community tensions, lack of clarity about communication channels and decision-making structures, and the stress and burnout that commonly accompany navigating the complex recovery process. Many participants expressed a desire to include fire survivors in LTRO leadership roles, as including survivors’ voices in decision-making was highlighted as key to serving the community equitably. However, they also noted concern that serving in this role would be traumatic for the survivors who had not yet had time to heal.

Participants had divergent perspectives, depending on which LTRO they led, about whether the standardized LTRO model [20] endorsed by FEMA and National Voluntary Organizations Active in Disaster (NVOAD) was the best fit for their community. Leaders of the longest operating LTRO in the study shared their perspective that this model was useful, and the structure of their organization still essentially matched that model. The smaller size of other LTROs’ communities and/or limited volunteers available to engage in LTRO leadership made staffing such a model unsustainable. In the words of one participant, whose LTRO initially formed in the traditional model,

*‘We’re supposed to have a public affairs committee. Our public affairs committee is the fact that the president and I both have the personal cell phone numbers of our two government representatives. […] We didn’t need a committee. […] It would have been nice. But in a town this size, this tapped out, it was never going to happen.’*

Several participants also noted that the FEMA- and NVOAD-endorsed LTRO model’s focus on individual service provision did not utilize the many pre-existing community organizations. They described their LTRO’s primary role as facilitating the collaboration of these various organizations that could each provide individual services to residents under the umbrella of recovery.

The uncertainty surrounding future LTRO funding was also reported as complicating LTRO functioning. Some LTROs lacked funding to hire an employee initially, which left an entirely volunteer leadership team to run an organization in what often became a second fulltime job. Participants reported hoping to hire more employees to support LTRO activities, but only had guaranteed funding for such positions for a year, making it difficult to attract candidates with the necessary skills. Some participants described worrying that funding agencies’ priorities could shift away from their organization and community as soon as the next disaster occurred. These financial uncertainties made establishing a stable governing structure for the organization extremely complex.

#### 3.2.3. Slow delivery of recovery resources

Participants identified a variety of delays in the delivery of key recovery resources. For instance, a delayed Major Disaster Declaration left several LTROs’ communities without much-needed resources to kickstart the recovery. State and local governments use FEMA public assistance as reimbursement for hazardous material cleanup costs, particularly important for wildfires that leave behind toxicants. In addition to funding, a Major Disaster Declaration typically brings technical support, both from FEMA and other agencies, that helps organize the response phase and facilitate the transition into recovery. Without this financial and technical support, necessary activities such as property cleanup were delayed. One participant described turning away volunteers who wanted to assist with property cleanup because the properties had yet to be inspected for toxic waste.

Even if the Major Disaster Declaration occurred quickly, key elements of individual assistance arrived very slowly. Placement of FEMA Transportable Temporary Housing Units [13], commonly referred to as ‘FEMA trailers,’ could be delayed up to nine months after the disaster. This left those in need of a FEMA trailer without this source of temporary stability for months. Due to the 18-month limit on FEMA trailer use, a timeline that began the day the wildfires received Major Disaster Declarations, a good portion of their time to actually live in the trailer evaporated. As one participant described it,

*‘We get four months use of these units […] And now we have these extensions, and you have these people living in limbo of, instead of knowing they have 18 months to work on their recovery, now they have to wonder every six months if they are. And then now we’re starting to charge them rent because we’ve exhausted what our normal extensions were.’*

The multi-round denial and appeal process that community members often endured to access the financial component of individual assistance also delayed the arrival of this resource.

In communities where utility companies bore responsibility for the fire, funds from pending settlements took many years to arrive. In turn, funders at Unmet Needs Roundtables, committees comprised of representatives of organizations that can provide financial, in-kind, or other resources to survivors whose recovery needs cannot be met through traditional programs [30], were often hesitant to provide financial support to survivors until they exhausted FEMA funding options, received pending settlement money, and utilized other community resources, in an attempt to be good stewards of donated dollars. However, due to the slow arrival of these resources and/or the length of time it took to reach a final funding decision, survivors were reported to remain in limbo for years before their unmet needs were addressed, greatly slowing their recovery process. Because of the rapidly increasing housing costs as the years passed, one participant also pointed out that,

*‘as people are waiting for these settlement funds, the ability of those settlement funds to complete their plan diminishes.’*

#### 3.2.4. The intertwined nature of community economic health and the built environment

Participants from all six LTROs expressed that a large proportion of the wildfire survivors were from at-risk groups (e.g., elderly, undocumented immigrants, those of lower socioeconomic status) whose source of affordable housing was destroyed by the fire. Rebuilding affordable housing stock will likely take years, if it can be replaced at all. Building apartments, usually the most efficient method of expanding affordable housing options, was described as inappropriate for their rural communities, whose residents often preferred to be away from other people, to live off the grid, or to have the additional flexibility that came with living outside of a more urban area. Building apartments also required infrastructure, such as a sewer system, that some communities currently lacked and had limited revenue/tax base to construct and maintain, especially post-wildfire. Participants also spoke more generally about the public revenue challenges communities faced after the wildfire, with residents displaced and no longer paying for public utilities that previously funded public services, such as trash pickup. This problem was exacerbated when greater numbers of residents permanently or temporarily moved away. The communities whose Major Disaster Declarations were delayed also lacked FEMA public assistance in the early phase of their recovery that would have replaced some of the lost funding for public services or rebuilding public infrastructure, further challenging the communities’ recoveries.

The disappearance of affordable housing meant that much of the workforce was forced to reside, at least temporarily, outside of the community. Without these residents, local businesses and industry lacked the workforce needed to rebuild and reopen. But residents also needed employment or other income-generating opportunities to be able to return to the community and remain stably housed. This created what one participant described as ‘a chicken and the egg’ problem. No residents mean no businesses, and no businesses mean limited economic opportunities for community members who return.

### 3.3. Facilitators to LTROs’ ability to meet community needs

#### 3.3.1. Collaboration with partners with a local presence

Each participant described numerous LTRO partners who supported their community’s recovery work. Organizations with a pre-disaster local community presence were particularly important LTRO partners. These agencies already had built trusting relationships with local community members and provided scaffolding into which disaster case management services could be integrated. They could also act as ‘boots on the ground’ in the local community, informing the LTRO about pockets of unmet need that might have otherwise been difficult to identify.

Close collaboration with community partners often enabled the LTRO to provide a coordinated entry point to various services, lessening the chance of service duplication or that community members might miss accessing a key recovery resource. Such collaboration also provided a mechanism for the LTRO to receive feedback from community members about barriers to accessing various recovery resources and to facilitate improvements in that access. One participant identified this as key to an equitable recovery process, saying,

*‘[…] we have this opportunity to work both at the grassroots level through our partnerships and also at the highest echelons of decision-making, we’re able to sew those two together. […] Whether or not we’re designing a system that’s equitable, if that’s not the experience on the ground, then we have work to do. And so building it again is that culture of quality improvement.’*

As this participant noted, the collaborative structure of the LTRO provided a forum to make recovery partners aware of any resource access barriers in the community and to incorporate solutions into high-level recovery planning decisions. Collaborative work with community partners also allowed LTROs to achieve greater impact with fewer funds. As one participant, whose LTRO underwent a structural transformation to a collaborative organizational model made up of committees of community partners, noted,

*‘[…] they’ve changed it to that collaborative model so that it wasn’t so staff-heavy [...] the more work we can do with less people, the more funding can go towards the Unmet Needs Roundtable and other programs that are going to help recovery.’*

#### 3.3.2. Flexibility in policies and procedures created by LTROs and government partners

Many participants identified the ability to adapt internal organizational policies and procedures, as well as flexibility in policies and procedures from government partners, as a key facilitator toward community recovery. Internal flexibility allowed LTROs to mitigate harmful impacts of the policies governing access to FEMA individual assistance and the disaster case management process described above. For instance, LTROs that controlled the disaster case management intake process could shift their policies to include additional community members, or leave the intake period open longer than originally intended, as some of the most vulnerable community members often did not request services until many years into the recovery process. LTROs could also adjust policies governing the funds available via the Unmet Needs Roundtable to better serve survivors whose needs did not fit the qualifications for other resources. As one participant said,

*‘As we’ve evolved, there are so many nuances and unmet needs that we’ve had to become more flexible. So what about the renter that takes his FEMA money and uses it to buy a piece of land hoping to rebuild, and none of the grants, federal or state, help that kind of a survivor, right? But yet, they’re trying to wisely make some decisions to recover.’*

Some LTROs did not control the disaster case management intake process, but still found creative ways to provide recovery resources to those without access to disaster case managers. As mentioned previously, leaders from one LTRO discovered that the disaster case management process in their community defined residency in a way that categorized many community members as ineligible. These leaders secured funding for community-based disaster case managers to serve these community members and present their cases to the Unmet Needs Roundtable, access to which is normally limited to those who have a disaster case manager.

When identifying how flexibility amongst government partners had facilitated their community recovery work, participants described how county governments gave the community a temporary reprieve from land use regulations and building code requirements, lowering the barrier to reconstruct homes built before these regulations were instituted. State government officials also adapted to unusual circumstances; for instance, when a Major Disaster Declaration was delayed, the state took the unprecedented step of activating SBA assistance prior to the declaration to help fill the recovery funding void this delay created. Another state’s officials designed a creative solution to address the logistical challenge of feeding families placed in non-congregate sheltering during the COVID-19 pandemic. Rather than hiring a corporate food service contractor, the state created a coalition of local restaurants whose businesses had been decimated by the pandemic, paying them to cook and deliver food to the families. This had the added benefit of creating food service job opportunities in the community.

The adaptability of FEMA representatives was a key facilitator for one LTRO’s community in particular, where a significant number of undocumented and mixed documentation status households were impacted by the wildfire. Because FEMA and Immigration and Customs Enforcement (ICE) are both located within the Department of Homeland Security, meaning the two agencies’ badges and uniforms resemble each other, members of these populations who were eligible for FEMA individual assistance were afraid to seek it. One participant described how FEMA representatives adapted to this concern:

‘*There were lots of questions about how undocumented or mixed-documentation-status families would be able to access individual assistance, and also whether or not they should […] So the experience on the ground is, ‘ICE is here. Don’t go anywhere near this.’ […] And so we were really fortunate that we had some very responsive FEMA partners on the ground who were like, ‘We’ll dress in plain clothes.’ ’*

The FEMA representatives also conducted outreach through local organizations that served these communities pre-disaster. These combined adjustments led to increased engagement of mixed documentation status households with FEMA, facilitating a more equitable distribution of individual assistance.

## 4. Discussion

A variety of factors impact LTROs’ ability to address rural communities’ social, economic and health needs following a wildfire. Barriers include factors external to the community (e.g., inflexible policies), internal to the community (e.g., restoration of the built environment), and organizational characteristics (e.g., LTRO organizational structures). On the other hand, extensive collaborations and the adaptability of LTROs and their partners supported LTROs’ ability to navigate around policies and procedures to meet community needs. Our findings highlight some of the pathways through which inequities in recovery resource access may occur, leaving vulnerable populations such as undocumented and mixed-documentation status households, those with precarious pre-disaster housing, and those with limited technological literacy to bear the worst of the wildfires’ long-term impacts. LTROs are aware of these disparities in disaster impacts and resource access, and described numerous strategies that they employed to mitigate these inequities.

The amount, timing, and accessibility of FEMA recovery resources greatly impact LTROs’ ability to support community members in securing post-disaster economic stability, housing, and other key social and economic resources that support health and wellbeing. Our results affirm the potential for FEMA policies to exacerbate community inequities, which has been explored in prior research [31, 32], correlating the distribution of FEMA individual assistance with increased wealth inequality [33] and documenting the increased post-disaster vulnerability of undocumented immigrants, who are excluded from individual assistance [34]. Prior studies also support our finding that extreme delays in the arrival of FEMA assistance and other recovery resources pose a major barrier for community recovery, both in the context of wildfires [3] as well as other types of major disasters [32]. Our findings also indicate that standard disaster case management models can exclude many community members who would benefit from access to this resource. Future research to understand how disaster case management models can contribute to or detract from equitable recovery resource access is warranted.

Increased cognizance of how their policies and standard operating procedures influence the recovery of communities’ social and economic infrastructure could greatly improve FEMA’s ability to support equitable community recovery. Our study participants offered practical reforms to FEMA policy to achieve this. Specifically, improvements to FEMA individual assistance could better facilitate the community recovery process. Two relatively low barrier changes that FEMA could implement would be to use a trauma-informed communication approach (e.g., remove language such as ‘eviction’ and ‘denial’) and to extend the length of time that community members can reside in FEMA trailers before they are forced to begin paying rent and/or leave. At a minimum, the 18-month timeline FEMA provides for trailer use could begin when the trailers are actually placed in the community and inhabited by residents, rather than the day the Major Disaster Declaration occurs.

Larger, structural changes to FEMA would facilitate broader access to individual assistance as well. Any real or perceived distance that FEMA representatives can create between themselves and the Department of Homeland Security, through uniform changes or by bureaucratically relocating the agency to another department entirely, would increase the comfort of communities wary of interacting with Homeland Security, such as mixed documentation status households, to seek FEMA assistance. Though more politically difficult to achieve, broadening access to FEMA individual assistance to include undocumented residents and those who have informal rental agreements or otherwise lack paperwork to prove their residency would facilitate a far more equitable recovery process. FEMA appears to be aware that prior restrictions on acceptable documentation to prove residency have excluded many community members, and as of September 2021, have expanded their list of proof of residence documents to include motor vehicle registrations, court documents, letters from local schools, federal or state benefit providers and social service organizations, signed statements from mobile home park owners, and self-certification for mobile homes and travel trailers [35]. These policy changes should be evaluated to determine whether they translate into improved access to FEMA assistance for vulnerable populations impacted by disasters.

The economic recovery barriers facing wildfire-impacted communities, such as a significant reduction in tourism in an area that relied on this as a revenue source or the destruction of affordable housing options for local workforces, are difficult challenges that may take many years for communities to address. Prior research also aligns with the finding that the damage to community economic health is a key barrier to post-wildfire recovery. These economic impacts have been characterized at the individual level, such as the large-scale loss of employment in the wake of a wildfire [3]; and at the community level, as wildfires have been shown to have lasting negative impacts on local municipality budgets, reducing available public service funding [36]. This economic damage is often inflicted on communities with limited pre-wildfire economic resources. Prior research confirms these findings beyond the communities represented in this research. For example, a study that examined wildfire frequency in California between 2000 and 2020 found that census tracts with higher proportions of vulnerable populations, such as the elderly and those of lower socioeconomic status, were disproportionately impacted [37].

In response to these challenges, LTROs can incorporate provision of economic resources as a core function. At least half of the LTROs included in the study were already doing this, either through engagement with local workforce service agencies or the inclusion of a specialized economic and workforce committee into their overall structure. Disaster case managers, who ideally have built personal rapport with their clients and already connect them with resources to meet other recovery needs, are naturally poised to serve as a liaison between clients and employment and/or other resources that can provide lasting economic support. Given the correlation between a stable source of income and housing stability [38], which was highlighted by participants, the incorporation of resources to address long-term economic needs is a necessary complement to LTROs’ critical work facilitating the return of community members to stable housing.

Despite widespread knowledge that the degree of collaboration between recovery organizations greatly impacts post-disaster recovery trajectories, effective collaboration remains difficult to implement [27]. Clarity of roles, trust between organizations, and governance structures that facilitate collaboration have been identified as key factors that impact ability to collaborate during the recovery process [27]. Our participants offered insights into how their own organizational structures transformed to facilitate better collaboration with community partners, demonstrating the specific ways that LTROs are successfully able to work collaboratively with partners towards community recovery, as well as the areas where that collaboration could be strengthened.

Many of the difficulties LTROs in the study faced were outside of the organizations’ control, but the LTROs’ ability to respond to some of these challenges in the earlier phases of the recovery process may have been inhibited by the internal growing pains they faced trying to create a functioning structure. Several participants highlighted that they wished a structure for the LTRO had existed in their community pre-disaster, so that they did not have to identify leaders and create a functional organization from scratch amidst the chaos post-wildfire. The longest functioning LTRO in the study served a community that had experienced several disasters since the initial wildfire that necessitated their formation, and had been able to use their existing structure to facilitate partnerships, recovery resources, and preparedness activities. This suggests that LTRO formation, and recovery planning more generally, could actually be a key disaster preparedness goal of communities, rather than being confined to the recovery phase of the disaster cycle. Preparedness phase LTRO formation would also allow adequate time to develop partnerships and make plans to address issues of equity without an unmet sea of post-disaster needs creating the pressure for quick, rather than thoughtful, action. In the words of one participant,

*‘This is the kind of work you have to do in non-emergency times. Because it’s all relationally driven, so I’m going to go to the people that I trust. And if everyone I trust is white and middle-aged and wealthy like I am, well, that’s my network […] I’m going to nurture those relationships [outside my network] in the calm. I don’t nurture them in an emergency.’*

### 4.1. Limitations

Our study is limited by a small sample of LTRO leaders. Though five participants were wildfire survivors themselves, this study does not comprehensively describe the perspectives of the community members served by the LTROs. The small sample size also limited our ability to systematically compare the communities served by participating LTROs based upon pre-disaster socioeconomic or demographic differences, or other factors such as the timing of the wildfire event that could influence vulnerability, donations, or the general functioning of an LTRO. Because some of the experiences the participants reflected on occurred several years ago, participants may have not completely or accurately recalled certain details. Our study used purposive sampling to identify prospective participants who could uniquely provide insights into the phenomenon of interest due to their lived experience and expertise. However, purposive sampling is a non-random sampling technique with the potential for selection bias.

## 5. Conclusion

Through semi-structured interviews with 18 LTRO leaders, this study provides insight into the barriers and facilitators of LTROs’ ability to meet post-wildfire, rural community needs. As a central coordinating point for recovery resources whose presence in a community can last upwards of five years, members of LTROs have deep insight into the factors that impact the community recovery process, and how these factors evolve throughout the multiyear recovery process. Our findings indicate that LTROs face many barriers in addressing community needs post-wildfire, including the policies that govern access to FEMA individual assistance and disaster case managers, whether managed by FEMA or other entities, the slow arrival of recovery resources and funds, the intertwined nature of community economic health and built environment restoration, and the challenge of forming a functional LTRO structure matched to community needs and capacity. However, participants also identified facilitators of LTROs’ work to community needs, including the ability of LTROs and their government partners to adapt policies and procedures, and close collaboration with other community organizations. This study’s findings suggest the need for policy improvements to promote more equitable access to recovery resources such as disaster case managers and FEMA individual assistance, that economic recovery should be a core LTRO function, and that recovery planning, including the formation of an LTRO, should be incorporated into community disaster preparedness activities. Future research should expand upon this exploratory study’s findings, focusing on these unique, community-based organizations in other communities and contexts.

## Data Availability

The data cannot be made publicly available upon publication because they contain sensitive personal information. The data that support the findings of this study are available upon reasonable request from the authors.

## Appendix A Semi-Structured Interview Guide

**Note:** ** is used to indicate priority questions for participants short on time

### Social Capital/Community Cohesion

****QUESTION 1:** ****Do you or did you ever live in a community that was/is served by the LTRO?**

****QUESTION 2: If the participant either lived or used to live in the community:** Can you tell me a bit about the community culture prior to the fire? (**Prompts:** In other words, how did they connect socially? Would you describe the community as ‘close-knit’ before the fire? Were there any pre-existing tensions?)

- ****Can you describe how the fire impacted the community culture?** Did this change throughout the recovery process?

****QUESTION 2: If the participant never resided in the community: **Can you tell me a bit about the community culture during the initial recovery process?** (**Prompts:** In other words, how did they connect socially? Would you describe the community as ‘close-knit’? Were there any tensions? Did this change throughout the recovery process?)

### Organizational Culture of LTRO

****QUESTION 3: To start, can you describe the LTRO’s primary goals?** (**Prompts:** What were the focuses of its community recovery activities? Was the focus on a unified community recovery plan? Serving individuals in their recovery? Both?)

- How did/have these goals and priorities shifted throughout the recovery process?

**QUESTION 4:** Can you describe the process the LTRO used for making important decisions? (**Prompts:** Did employees, all board members, only those present at a meeting, etc give input? How were any disagreements resolved?)

### Community Involvement

**QUESTION 5:** In the survey, you indicated that the LTRO uses/used ***(insert communication methods here)*** to communicate with community members. What kinds of information is generally communicated by each of these methods?

- Are/Were some methods of communication more effective than others, and if so why? How have communication strategies shifted throughout the recovery process?
  * **If the community has residents who spoke a 1st language other than English:** Can you describe any approaches the LTRO takes/took to communicate with community members unable to speak or understand English? How adequate are/were the language and translation resources that the LTRO has/had access to, if any?

****QUESTION 6:** You also indicated that the LTRO uses/used ***(insert engagement methods here)*** to engage community members in the LTRO’s recovery plans and activities. **Could you elaborate a bit on how each of these methods is/was used to engage the community in the LTRO’s recovery plans and/or activities?**

### LTRO Resources

**QUESTION 7:** You mentioned the LTRO had/has ***(insert funding sources here)*** as sources of funding. What was that funding primarily used for? Were there any challenges in accessing or administering this funding?

****QUESTION 8: If the fire received a Presidential Disaster Declaration: Can you talk a bit about how the Presidential Disaster Declaration determination and process impacted the LTRO’s work?** (**Prompts:** individual, public, hazard mitigation, or multiple types)

- **If individual assistance was available:** Can you describe any challenges or successes community members have/had accessing individual assistance?
- **If public assistance was available:** Can you describe any challenges or successes your organization or response partners have/had faced using or accessing public assistance?

****QUESTION 8: If the fire did not receive a presidential disaster declaration:** I saw in my background research that ***(insert specific fire name)*** did not receive a Presidential Disaster Declaration. **Can you talk a bit about how that impacted the LTRO’s work?** (**Prompts:** impact on LTRO operations, impact on individual community members, impact on recovery of public/shared spaces)

### LTRO Partnerships

****QUESTION 9: What organizations did/does the LTRO collaborate with, and how?** (**Prompts:** recovery-specific organizations, pre-existing community organizations, local government)

### Resources Offered to the Community

****QUESTION 10: Can you describe any other resources offered to individual community members by the LTRO or its partners, beyond those you have already discussed?** (**Prompts:** to those who either lost housing/experienced damage, lost income or employment, and/or experienced physical or mental health impacts caused by the fire?)

**QUESTION 11:** Did the LTRO or its partners support rebuilding community spaces, such as ***(reference any community structures, parks, government buildings, etc impacted by the fire),*** beyond those examples you have already discussed?

****QUESTION 12: Could you describe any significant unmet needs that remain in the community? What are/were the challenges in meeting these needs?**

### Accessibility & Equity of Resources

****QUESTION 13: Can you describe any policies or plans the LTRO has/had to ensure all community members and/or communities had equitable access to recovery resources?**

- Can you describe how the LTRO discussed and/or defined ‘equity’? (**prompts:** was it thought about in terms of race/ethnicity, socioeconomic status, preferred language, geographic location, etc.)

### Closing Questions

**QUESTION 14:** Reflecting back on what you have shared with me already, it sounds as though ***(insert challenges the participant has mentioned)*** were challenging for the LTRO/community during the early stages of the recovery process, and as the recovery progressed ***(summarize new challenges or changes to early challenges)***. *(**Insert unmet needs)*** remain an unmet need in the community. Is there anything you would like to add? You can elaborate on challenges we have already discussed, or tell me about another important challenge in the community recovery process that we have not yet discussed.

**QUESTION 15:** You mentioned that ***(insert successes the participant has mentioned here)*** as aspects of the LTROs work/community that were successful early in the recovery process, and that later in the recovery process ***(summarize new successes or changes to early successes)***. Is there anything you would like to add? You can elaborate on successes we have already discussed, or tell me about another important success in the community recovery process that we have not yet discussed.

**Closing Remarks:** That concludes the questions I had planned to ask you. Is there anything else you’d like to add before we wrap up? Do you have any additional questions for me?

## Appendix B Qualitative Codebook

**Table:**

Code ^a^ ^,^ ^b^	Definition
**History**	Any information describing the pre-disaster history of the community or the interviewee’s professional or personal history, especially with the community/communities served by the LTRO
**COVID-19**	Any information describing how the COVID-19 pandemic and associated societal responses to the pandemic impacted the progression of response and/or recovery activities
**Community Recovery as a Shared Value (RWJF)**	Statements describing the extent to which local community members and the LTRO facilitated and participated in a collective vision of community recovery
Collective Recovery Mindset	Statements describing the extent to which community recovery was viewed as a collective, intertwined process by both community members and LTRO leadership, and the willingness of community members and/or LTRO leadership to collaborate with each other or amongst themselves (e.g. Did interpersonal conflicts impact the LTRO’s work?; Was there an understanding that each community member’s recovery benefited the recovery of the community as a whole?; Was the recovery public and/or community resources seen as important to individual recovery?)
Civic Engagement	Statements describing the extent of community members’ engagement and collaboration in the recovery process, as well as the LTRO’s efforts to engage the community members; this includes participation in LTRO activities or leadership as a community member (e.g. collective efforts to clean up, attendance to community meetings about recovery, community members involvement with LTRO)
Sense of Community	Statements describing the evolution of community cohesion pre-disaster and post-disaster throughout the recovery process
**Cross-Sector Collaboration (RWJF)**	Statements describing the extent of cross-sector collaboration throughout the community recovery process, such as the number and quality of partnerships and financial, time, and personnel resources invested in collaboration (e.g. between local, state, and federal government, response and recovery organizations, local community organizations, and the LTRO)
**Health and Equity of Communities (RWJF)**	Statements describing social determinants of health or government/public policy’s impact on social determinants of health in the community/communities served by the LTRO, both pre- and post-disaster (e.g. the availability of affordable housing, employment and education opportunities, transportation, discrimination, environmental pollution, healthcare access, etc)
Built Environment	Statements describing aspects of the community/communities’ built environment (e.g. housing, infrastructure, parks, etc.); can be either pre- or post-disaster
Social and Economic Environment	Statements describing aspects of the community/communities’ social and/or economic environment (e.g. employment opportunities, educational opportunities, aspects of culture, funding for public services, political leanings of community members, etc.); can be either pre- or post-disaster
Policy and Governance	Statements describing laws, policies, and/or standard procedures’ impact on community recovery and equity in recovery, including the policies of the LTRO (e.g. land use or building regulations, organizational policies about who qualified for access to a particular resource, etc)
**Integration of Recovery Systems (RWJF)**	Statements describing the accessibility, degree of coordination, and appropriateness of the manner in which recovery resources were provided; can apply to integration at the organizational, community, or societal levels (e.g. whether communication about resources was available in community members’ preferred language, if resources provided to community members matched their stated needs, whether community members had a clear access point to all available resources)
Balance and Integration	Statements describing the extent to which the organizations providing recovery resources integrated their activities to provide coordinated recovery services to impacted community members; likely to be co-coded with Cross-Sector Collaboration code above
Consumer Experience and Quality	Statements describing if and/or how recovery resources were provided in a manner that considered the needs and preferences of recipients; including the availability of communication in the preferred language of recipients
**Barrier (co-code)**	Statements describing any barrier to community recovery encountered by the LTRO or its partners at any point during the recovery process; can be explicitly stated by interviewee or inferred from interviewee’s description of events; co-coded with RWJF domain codes above
Organizational Level (co-code)	Barrier occurring at the organizational level (i.e. within the LTRO); co-coded with RWJF domain codes above
Community Level (co-code)	Barrier occurring at the community level (i.e. within the control of the community or communities served by the LTRO); co-coded with RWJF domain codes above
Societal Level (co-code)	Barrier occurring at the societal level (i.e. outside the control of individual community members, community organizations, or local community government); co-coded with RWJF domain codes above
**Facilitator (co-code)**	Statements describing any facilitator to community recovery encountered by the LTRO or its partners at any point during the recovery process; can be explicitly stated by interviewee or inferred from interviewee’s description of events; co-coded with RWJF domain codes above
Organizational Level (co-code)	Facilitator occurring at the organizational level (i.e. within the LTRO); co-coded with RWJF domain codes above
Community Level (co-code)	Facilitator occurring at the community level (i.e. within the control of the community or communities served by the LTRO); co-coded with RWJF domain codes above
Societal Level (co-code)	Facilitator occurring at the societal level (i.e. outside the control of individual community members, community organizations, or local community government); co-coded with RWJF domain codes above
**Key Quote**	Statements by participants that should potentially be highlighted in future papers and presentations; can be great examples of the other codes above, or simply powerful statements or recommendations

^a^ Parent codes are bolded; associated child codes are located below bolded parent codes.

^b^ Codes adapted from the Robert Wood Johnson Foundation’s Culture of Health Action framework are denoted with ‘RWJF’.
